# *In Vitro* and *In Vivo* Insights into a Broccoli Byproduct as a Healthy Ingredient for the Management of Alzheimer’s Disease and Aging through Redox Biology

**DOI:** 10.1021/acs.jafc.3c05609

**Published:** 2024-03-05

**Authors:** María D. Navarro-Hortal, Jose M. Romero-Márquez, M. Asunción López-Bascón, Cristina Sánchez-González, Jianbo Xiao, Sandra Sumalla-Cano, Maurizio Battino, Tamara Y. Forbes-Hernández, José L. Quiles

**Affiliations:** † Department of Physiology, Institute of Nutrition and Food Technology “José Mataix Verdú”, Biomedical Research Centre, University of Granada, 18016 Armilla, Spain; ‡ Research and Development Functional Food Centre (CIDAF), Health Science Technological Park, Avenida del Conocimiento 37, 18016 Granada, Spain; § Sport and Health Research Centre, University of Granada, C/Menéndez Pelayo 32, 18016 Granada, Spain; ∥ Department of Analytical Chemistry and Food Science, Faculty of Food Science and Technology, University of Vigo, Ourense Campus, E-32004 Ourense, Spain; ⊥ Research Group on Foods, Nutritional Biochemistry and Health, Universidad Europea del Atlántico, Isabel Torres, 21, 39011 Santander, Spain; # Department of Health, Nutrition and Sport, Iberoamerican International University, Campeche 24560, Mexico; ∇ Department of Clinical Sciences, Polytechnic University of Marche, 60131 Ancona, Italy; ○ International Joint Research Laboratory of Intelligent Agriculture and Agri-Products Processing, Jiangsu University, Zhenjiang 212013, China

**Keywords:** sulfur compounds, amyloid-β, hyperphosphorylated tau, SKN-1/Nrf2, heat shock protein, lipofuscin

## Abstract

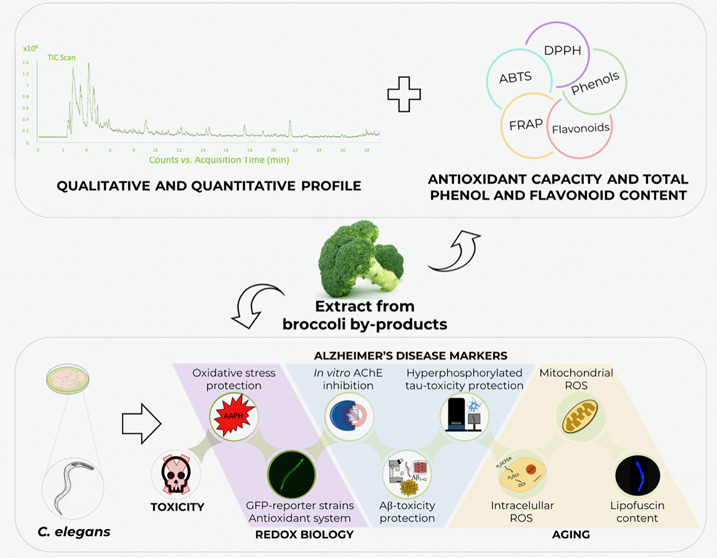

Broccoli has gained popularity as a highly consumed vegetable due to its nutritional and health properties. This study aimed to evaluate the composition profile and the antioxidant capacity of a hydrophilic extract derived from broccoli byproducts, as well as its influence on redox biology, Alzheimer’s disease markers, and aging in the *Caenorhabditis elegans* model. The presence of glucosinolate was observed and antioxidant capacity was demonstrated both *in vitro* and *in vivo*. The *in vitro* acetylcholinesterase inhibitory capacity was quantified, and the treatment ameliorated the amyloid-β- and tau-induced proteotoxicity in transgenic strains via SOD-3 and SKN-1, respectively, and HSP-16.2 for both parameters. Furthermore, a preliminary study on aging indicated that the extract effectively reduced reactive oxygen species levels in aged worms and extended their lifespan. Utilizing broccoli byproducts for nutraceutical or functional foods could manage vegetable processing waste, enhancing productivity and sustainability while providing significant health benefits.

## Introduction

Broccoli (*Brassica oleracea* var. italica) is a plant belonging to the *Brassicaceae* family that has gained significant popularity as a highly consumed vegetable, becoming a staple in diets and markets. However, the process of harvesting broccoli generates a substantial number of residues and byproducts, including leaves, stalks, and damaged or low-marketable florets, which seemingly hold little or no economic value.^1^ In fact, those materials constitute more than 95% of the harvested material.^2^ The agri-food industry produces numerous byproducts that can serve as valuable sources of nutrients and potentially functional ingredients, offering opportunities to create value-added products.^3^ Broccoli and its byproducts possess a high nutritional value, due to its wealth in dietary fiber, vitamins (A and C), and essential mineral nutrients (calcium and iron) in its composition. Moreover, they contain bioactive compounds such as numerous phenolic compounds (especially flavonoids and hydroxycinnamic acids) and glucosinolates.^1,2^ Different functions have been attributed to this vegetable and its biological properties may contribute to improve well-being for consumers, like reducing the risk of chronic diseases including neurodegenerative and promoting health status.^2^

An important challenge with great scientific interest due to its social, economic, and health relevance is the aging of the population. The projected number of individuals over 80 years or older is anticipated to triple between 2020 and 2050, reaching approximately 426 million.^4^ Aging is not a disease, but a physiological process that instead is closely related to the so-called age-related diseases, including Alzheimer’s disease (AD). AD is the most prevalent neurodegenerative disorder, with cognitive impairment and memory loss as the main symptoms. There are several hypotheses about AD etiology, including the oxidative stress theory, cholinergic hypothesis, amyloid cascade hypothesis, and tau protein hyperphosphorylation hypothesis.^5^ Oxidative stress has been recognized as an early feature in the AD physiopathology, leading to a compensatory aggregation of amyloid-β (Aβ) and hyperphosphorylated tau.^6−8^ Furthermore, the senile depositions in the form of Aβ plaques and neurofibrillary tangles of hyperphosphorylated tau protein have been correlated with the elevated levels of reactive oxygen species (ROS) and oxidative stress markers such as lipid peroxidation products,^9,10^ and with the decrease in the antioxidant defense^10,11^ in AD patients. The cumulative impact of increased oxidative stress due, in turn, to Aβ plaques and hyperphosphorylated tau deposits can lead to cellular deterioration, potentially resulting in cell death or apoptosis.^12,13^ As the aging population grows, AD incidence does too. The lack of effective treatments makes necessary the exploration of strategies to prevent or slow neurodegeneration. Both aging and AD are multifactorial phenomena influenced by redox biology and nutrition, which play decisive roles in their development. This is why foods or food byproducts such as broccoli could serve as excellent approaches to address these challenges.

Therefore, the present study aimed to explore bioactive compounds profile and amount as well as the total antioxidant capacity of a hydrophilic extract obtained from broccoli byproducts. Additionally, *Caenorhabditis elegans* has been used to *in vivo* assess the potential toxicity and the effect on protein-induced alterations associated with AD such as that caused by Aβ and tau aggregation as well as to deepen inside the molecular bases for any observed effect.

## Materials and Methods

### Plant Material and Extraction Process

Broccoli (*B. oleracea* L. var. Italica L.) byproducts extract (BRO) was provided by Ingredalia S.L. (Navarra, Spain). Broccoli florets and stems byproducts, harvested from November to May, were used. For extraction, 1.5 kg of those byproducts were mixed with 1 L of solvent extraction (ethanol/water 80:20, v/v) in a reactor. After 60 min of extraction at 30 °C, physical filters (sleeve filters) were used to remove solids: it was performed a first filtration with a pore size of 10–25 μm, and a second filtration of 1 μm. Then, it was concentrated under vacuum. The extraction yield was 4%. The dried extract was stored in aliquots at −80 °C and conveniently diluted in Milli-Q water for use.

### Characterization of the Extract

#### Evaluation of the Total Antioxidant Capacity (TAC)

The TAC evaluation was performed by 2,2-diphenyl-1-picryl-hydrazyl-hydrate (DPPH), 2,2′-azino-bis(3-ethylbenzothiazoline-6-sulfonic acid) (ABTS) radical assay, and ferric reducing antioxidant power (FRAP) techniques as previously described.^14−16^ Wavelengths used were at 515, 734, and 593 nm for DPPH, ABTS, and FRAP, respectively, and a Synergy Neo2 microplate reader (Biotek, Winooski, Vermont, USA) was used. Results were expressed as μmol trolox equivalent (TE)/g of dry extract (DE).

#### Total Phenolic Content (TPC) and Total Flavonoids Content (TFC) Measurement

The Folin–Ciocalteu method was performed to determine TPC,^17^ and TFC quantification was performed as previously described.^18^ The absorbance was measured at 760 and 510 nm, respectively, using a Synergy Neo2 microplate reader (Biotek, Winooski, Vermont, USA). For TPC, gallic acid was used as standard and for TFC, catechin was used. Results were expressed as mg of gallic acid or catechin equivalent/g DE.

#### Chromatographic Operating Conditions for Identification and Quantification of Individual Compounds

*S*amples were analyzed as described.^19,20^ Briefly, the extract was analyzed in triplicate using an Agilent 1260 series liquid chromatograph. For separation, an Agilent Zorbax Eclipse Plus C18 column with dimensions of 4.6 mm × 150 mm and a particle size of 1.8 μm was used. Mobile phases used were water with 0.1% formic acid as phase A and acetonitrile with 0.1% formic acid as phase B, with the following gradient: 0 min, 5% phase B; 20 min, 20% phase B; 25 min, 50% phase B; 33 min, 95% phase B, and finally, 7 min conditioning cycle. The flow rate was 0.5 mL/min, the column was maintained at 25 °C, and 5 μL of the sample was injected. Detection was performed with an Agilent 6540 Ultra High Definition (UHD) Accurate Mass Q-TOF detector equipped with a dual ESI Jet Stream interface. Detection by QTOF was performed in positive ionization mode, in a mass range of 50–1700 *m*/*z*. Ultrapure N2 was used as ionization and drying gas at a temperature of 325 and 400 °C, respectively, and flows of 10 and 12 L/min, respectively. Other parameters used were capillary voltage, 4000 V; N2 pressure in nebulizer, 20 psig; Q1 voltage, 130 V; nozzle voltage, 500 V; skimmer, 45 V, and octopole 1 RF, 750 V. The analysis was performed with the continuous ionization of the trifluoroacetate anion (112.985587 *m*/*z*) and a hexakis (1*H*,1*H*,3*H*-tetrafluoropropoxy) phosphazine adduct (1033.988109 *m*/*z*) with the aim of recalibrating each mass spectrum acquired during the analysis. MS/MS analyses were performed in automatic fragmentation mode, isolating and fragmenting the two most intense mass peaks, with the following collision energy values: 10, 20, and 40 eV. MS/MS data were acquired using the centroid mode at a rate of 2.5 spectra/s in the extended dynamic range mode (2 GHz). All data acquisition operations were controlled with Masshunter workstation software version B.06.00 (Agilent Technologies). The main compounds in the extract were automatically detected using a compound extraction algorithm based on molecular feature detection, and the resulting peaks were filtered with a relative volume threshold of 0.3% as well as those that appeared in the solvent blank. The compounds detected by this algorithm were tentatively identified, whenever possible, with the help of compound databases (SciFinder, HMDB, Metlin, etc.) and scientific literature related to vegetables, based on the molecular formula obtained from the exact mass and isotopic distribution data to the retention times and fragmentation patterns recorded.

#### *In Vitro* Assay of the Acetylcholinesterase (AChE) Inhibitory Activity

Ellman’s modified method was used.^21^ AChE activity was determined using 150 μM 5,5′-dithiobis(2-nitrobenzoic acid) (DTNB), 150 μM acetylthiocholine iodide (ATCh) (substrate), and 10 mU/mL AChE (in 50 mM Tris-HCl buffer, pH 8.0). AChE was incubated in 96-plate wells with DTNB and different concentrations of the extract, the inhibition control physostigmine (PHY), or Milli-Q water (positive control of AChE activity) for 15 min at 30 °C. Then, the substrate was added and absorbance changes at 405 nm for 25 min at 30 °C were measured. AChE inhibitory activity was presented as percentage (%) of inhibition compared with the positive control. The concentration of the extract causing 50% inhibition of AChE activity (IC_50_) was determined by regression analysis.

### *C. elegans* Experiments

#### *C. elegans* strains and maintenance

*C. elegans* strains used in this work included N2 Bristol, LD1 (ldIs7 [skn-1b/c::GFP + rol-6(su1006)]), TJ356 (zIs356[daf-16p::daf-16a/b::GFP + rol-6(su1006)]), TJ375 (gpIs1[hsp-16.2::GFP]), OS3062 ([myo-2p::hsf-1 + hsp-16.2::GFP + hsp-16.41::GFP + rol-6(su1006)]), CF1553 (mu1s84[pAD76(sod-3::GFP) + rol-6(su1006)]), CL2166 (dvIs19 [(pAF15)gst-4p::GFP::NLS] III), CL4176 (dvIs27 [myo-3p::A-β (1–42)::let-851 3′UTR) + (rol-6(su1006)] X), CL802 (smg-1(cc546) I; rol-6(su1006) II), and BR5706 (byIs193 [rab-3p::F3(delta)K280 + myo-2p::mCherry]. bkIs10 [aex-3p::hTau V337 M + myo-2p::GFP]). All of them were maintained in an incubator (VELP Scientifica FOC 120 E, Usmate, Italy) at 20 °C, except for CL4176 and CL802 that were kept at 16 °C. Worms were cultured on plates with nematode growth media (NGM) spread with *Escherichia coli* OP50, which was used by the worms as a source of food. Both the worms and bacteria were obtained from the Caenorhabditis Genetics Center (CGC) (Minneapolis, Michigan). Age-synchronized nematodes were used for all of the experiments, unless otherwise specified, obtained from gravid hermaphrodite adults treated with bleaching solution (0.5 N NaOH in 20% bleach).

#### Lethality Test

The death rate of N2 worms subjected to BRO (0, 100, 500, 1000, 5000, 7500, and 10 000 μg/mL) was used to assess acute toxicity in a concentration–response curve. L3 larvae were exposed to treatment for 24 h at 20 °C without food. Then, survival % was calculated using a microscope (Motic Inc., Ltd., Hong Kong, China). Each independent assay consisted of at least three NGM plates with at least 10 worms each.

#### Egg Viability Evaluation

Gravid N2 nematodes cultivated in standard conditions were subjected to synchronization, and the obtained eggs were placed in plates containing *E. coli* OP50 plus the treatments (0, 100, 500, 1000, 5000, 7500, and 10 000 μg/mL). After 24 h, the number of larvae was counted using a microscope. At least 40 eggs per group were used. Data were presented as the percentage of hatched eggs per group.

#### Lifespan Curves

N2 strain was used to perform survival curves in order to evaluate the potential long-term toxic effect of BRO.^22^ One hundred and twenty L3 synchronized worms were placed on fresh plates containing the treatments or the control plus *E. coli* OP50 and maintained at 20 °C. The concentrations used were 100, 500, 1000, 5000, 7500, and 10 000 μg/mL. The compound 5-fluoro-2′deoxyuridine (FUDR) (Sigma-Aldrich, St. Louis, Missouri) was used to prevent egg-laying during the fertile phase. The experiment commences at the L3 because this stage is considered ideal due to the FUDR embryotoxicity and the optimal efficacy of FUDR requires its application prior to the reproductive development of the worms. Worms were then scored for survival every day and transferred to fresh plates twice per week. Death was recorded when no response to a mechanical stimulus was observed. Animals that were removed from the dish or dead from progeny in utero were not included in the death count (censored). For each dosage, Kaplan–Meier curves were presented.

#### Measurement of Intracellular Reactive Oxygen Species (ROS) Content

N2 synchronized eggs were placed on plates with or without the treatments (0, 100, 500, and 1000 μg/mL) and incubated for 48 h at 20 °C. After that, all groups were washed with M9 medium and exposed (except the CTL basal group) to 2.5 mM 2,2′-azobis-2-amidinopropane dihydrochloride (AAPH) for 15 min. Then, the AAPH was removed by wash with M9, and worms were incubated for 2 h with 25 μM dichlorodihydrofluorescein diacetate (DCFDA) at 20 °C. A Multi-Range Large Particle Flow Cytometer Biosorter (Union Biometrica, Massachusetts, USA) was used to measure the fluorescence intensity of, at least, 300 worms per group. ROS content was estimated as a percentage of control of average yellow fluorescence intensity.

#### Redox Biology-Related Genes Measured in Green Fluorescent Protein (GFP)-Reporter Transgenic Strains

Transgenic LD1 and TJ356 nematodes express the fusion of the genes encoding the transcription factors SKN-1/Nuclear factor erythroid 2-related factor 2 (Nrf2) and DAF-16/Forkhead box transcription factors class O (FOXO), respectively. TJ375 and OS3062 worms express the *hsp-16.2* and the combination of *hsf-1:hsp-16.2:hsp-16.41* genes. The *sod-3* gene is expressed in the transgenic CF1553, and the *gst-4* in the strain CL2166. In all of them, the gene is fused with the GFP. For all strains, synchronized eggs were placed on control plates or plates containing BRO at concentrations of 100, 500, and 1000 μg/mL and maintained for 48 h. Subsequently, nematodes were immobilized on glass slides containing sodium azide to limit their movement. Worm images were captured with the 10× objective for all strains, except for TJ375 and OS3062, which were observed using 40× magnification. A Nikon epi-fluorescence microscope (Eclipse Ni, Nikon, Tokyo, Japan) equipped with a Nikon DS-Ri2 camera (Tokyo, Japan) and the GFP filter was utilized. NIS-Elements BR software (Nikon, Tokyo, Japan) was used for analysis. For the TJ356 strain, a semiquantitative scale was applied, assigning a value of “1” to worms exhibiting cytosolic expression of DAF-16::GFP, “2” to those with intermediate status, and “3” to worms showing nuclear localization. Then, the results were normalized and expressed as percentage to the control group. The fluorescence intensity of the entire worm was measured for LD1, CF1553, and CL2166. The area anterior of the pharyngeal bulb was measured for HSP16.2::GFP and HSF-1:HSP-16.2:HSP-16.41::GFP in TJ375 and OS3062 worms, respectively.

#### Paralysis Assay

The paralysis assay was conducted following the methodology already described.^23^ The CL4176 strain harbors a temperature-sensitive mutation that induces the expression of human Aβ_1–42_ peptide in muscle cells, leading to paralysis in the nematode. Synchronized eggs from CL4176 were distributed onto plates containing different concentrations of the extract (0, 100, 500, and 1000 μg/mL) along with *E. coli* OP50 as a food source. Nonparalyzable strain CL802 was used as a negative control in the experiment. Plates were incubated at 16 °C for 48 h and then shifted to 25 °C to induce Aβ expression. Paralysis was assessed from 20 to 32 h after temperature shifting, with each group and replicate including at least 25 worms. Results are presented as curves depicting the percentage (%) of nonparalyzed worms over time.

#### Aβ Plaques Staining

Thioflavin T dye was used with the aim of visualizing Aβ aggregates in the CL4176 strain as described.^14^ Worms and plates were treated as for the paralysis assay and, approximately at the time for 50% of paralysis of the nontreated group, the nematodes were collected by wash with M9. Then, they were fixed by using 4% paraformaldehyde (pH 7.4) at 4 °C for 24 h, followed by the application of permeabilization buffer (5% β-mercaptoethanol, 1% Triton X-100, and 125 mM Tris, pH 7.4). After 24 h at 37 °C, the buffer was removed by washing with M9 and 0.125% thioflavin T was used for the staining of the samples for 30 min. Subsequently, they were distained with sequential ethanol washes (50%, 75%, 90%, 75%, and 50% v/v, each one for 2 min). Thioflavin T-stained worms were visualized using a Nikon epi-fluorescence microscope (Eclipse Ni, Nikon, Tokyo, Japan) at 40× magnification. Images were captured with a Nikon DS-Ri2 camera (Tokyo, Japan) using the GFP filter.

#### Tau Proteotoxicity Assessment

BR5706 strain, characterized by pan-neuronal expression of Tau protein aggregates, displayed locomotion defects upon reaching adulthood. After incubating BR5706 worms with 100, 500, and 1000 μg/mL or without treatments for 72 h at 20 °C, animals were induced to swim to assess their movement. WormLab Imaging System (MBF Bioscience, Williston, Vermont, USA) was utilized to record, track, and analyze worm movement, focusing on swimming speed and activity index as representative locomotive behavior parameters.

#### Silencing of Targeted Genes by RNA Interference (RNAi) Technology

The RNAi technology was employed to inhibit the expression of target genes in both the paralysis test and the tau proteotoxicity evaluation test. *E. coli* HT115 expressing DAF-16/FOXO, SOD-3 (Cultek SL, Madrid, Spain), SKN-1/Nrf2, and HSP-16.2 (Sources BioScience, Nottingham, UK) dsRNA were spread on NGM plates. Isopropyl β-d-1-thiogalactopyranoside (IPTG) (1 mM) and carbenicillin (25 μg/mL) were added to the medium. L3–L4 synchronized worms (F0) were placed into the RNAi plates and, from them, eggs (F1) were isolated. Those eggs belonging to CL4176 and BR5706 were placed into the plates containing treatments with each RNAi and a plate without the RNAi. From this step, the experiments were carried out as explained above.

#### Measurement of Intracellular and Mitochondrial ROS Content in Aged Nematodes

**Figure 1:**
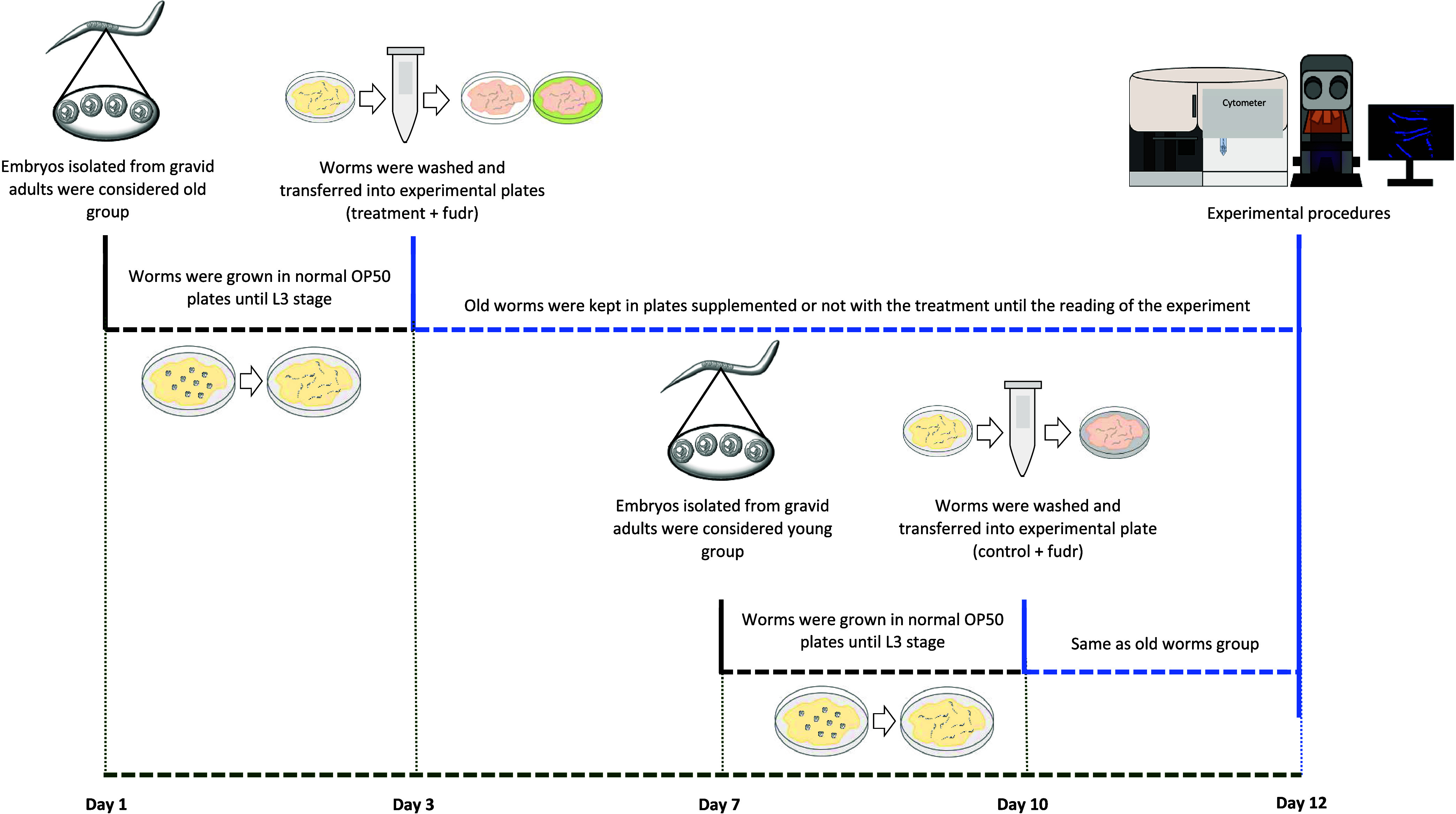
Experimental design for *in vivo* aging experiments.

The following protocols, represented in a scheme gathered in Figure 1, were applied to young (5 days old) and aged (12 days old) worms in a baseline situation. L3 stage-synchronized N2 nematodes were exposed to 100 μg/mL BRO or the respective control. Worms were incubated at 20 °C until the read-out point. After that time, intracellular ROS content was measured using DCFDA, as previously described for oxidative stress conditions, and the mitochondrial ROS content was measured using 10 μM Mitotracker Red CM-H2 XRos dye mixed with dead *E. coli* OP50 as a food source. A group of young worms was used as control. To avoid eggs laid during the fertile phase, 15 μg/mL FUDR (Sigma-Aldrich, St. Louis, Missouri) was applied. Furthermore, the animals were moved to new plates with fresh treatment and food twice per week in the case of the aged groups, and once for the young group. Fluorescence intensity was measured using Biosorter (Union Biometrica, Massachusetts). At least 300 worms per group were used. Results were expressed as the percentage of control using the mean of the yellow or red fluorescence intensity for total or mitochondrial ROS content, respectively.

#### Quantification of Lipofuscin Content

Protocol for young and aged worms was followed as explained in the previous section. Young and old nematodes were treated with 100 μg/mL BRO and the control group was mounted on glass slides with M9 medium and sodium azide. Fluorescence images were captured using an epi-fluorescence microscope (Eclipse Ni, Nikon, Tokyo, Japan). Images were acquired with a 10× objective lens and the autofluorescence of the worms was analyzed using the software NIS-Elements BR (Nikon, Tokyo, Japan).^22^ A minimum of 30 worms per group were measured. Results are the percentage to the control for the average of total blue (DAPI) fluorescence intensity for each worm.

#### Statistical Analysis

The normality of variables was assessed by the Kolmogorov–Smirnov test, and the homogeneity of variance was examined with the Levene test. Non-normally distributed variables were analyzed by using nonparametric tests, while normally distributed variables were subjected to *t*-student, or one-way ANOVA followed by Bonferroni *post hoc* tests. The Kruskal–Wallis and Mann–Whitney-U tests were applied for non-normally distributed variables. Data were presented as mean ± standard error of the mean (SEM) from at least 3 independent experiments, unless otherwise specified. Statistical significance was considered at *p* < 0.05. Survival analysis for lifespan curves was conducted by the Log-rank test. The data visualization and representation were done using the software Microsoft Excel 365 (Washington, USA). All statistical analyses were performed using IBM SPSS 24.0 (Armonk, New York).

## Results and Discussion

### Characterization of the Broccoli Byproduct Extract

**Figure 2:**
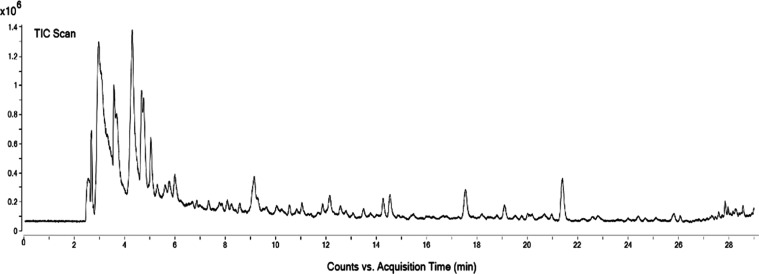
Positive chromatogram of broccoli byproduct extract (BRO).

Consumption of broccoli and its compounds has been linked to several benefits for health.^24^ These effects are often attributed to the abundance of micronutrients and phytochemicals in broccoli. Therefore, the extract from broccoli byproducts investigated in the present study was characterized. Concerning TAC, results were 219 ± 6.30 μM TE/g DE for FRAP, 103 ± 12 μM TE/g DE for DPPH, and 311 ± 9.20 μM TE/g DE for ABTS. Regarding TPC and TFC, it was found 34.4 ± 2.05 mg gallic acid/g DE and 3.87 ± 0.60 mg catechin/g DE, respectively. TPC, TFC, and TAC in broccoli can vary due to factors like harvest time, cultivar, plant part used, and extraction process, leading to differences in the literature. For instance, TFC of our BRO was found to be lower^25^ or within the same range^26^ compared with reported values. Similarly, TPC data from our extract was lower,^25^ similar,^26^ or much higher^27^ compared with other studies. TAC measurements with the ABTS test aligned with results obtained from a methanolic extract of broccoli florets.^27^ The positive chromatogram obtained from the analysis of BRO by HPLC-ESI-QTOF-MS/MS is shown in Figure 2, and the main identified compounds are listed in Table 1. Thirty-four compounds were identified with the main classes being amino acids and derivatives, carbohydrates and derivatives (including the sulfur compounds glucosinolates), carboxylic acids and derivatives (citric acid), and hydroxycinnamic (syringic acid glucuronide) and hydroxybenzoic (chlorogenic acid) acids. Concentrations of identified compounds are listed in Table 1. It should be noted that, given the available information, the precise identification of the compounds as either caffeoylquinic acid or chlorogenic acid cannot be conclusively determined. However, these two compounds are the most plausible candidates based on the data. This is reflected in their repeated listing in Table 1. Among the most abundant compounds, uridine (31.7 ± 0.3 mg/g), dihydroxy-dimethoxy-benzoxazinone (20 ± 1 mg/g), citric acid/isocitric acid (19 ± 1 mg/g), and methoxy tyrosine (16 ± 1 mg/g) stand out. Quantification of glucosinolates, which are typical compounds present in broccoli, was found to be 0.79 ± 0.02 mg/g. Notably, our extract contained chlorogenic acid, which was also present in other broccoli-related samples in higher concentrations.^26^ Additionally, one sulfur compound classified as glucosinolate derivate was detected in the sample. Differences in terms of TAC and composition between our extract and those characterized by other authors could be attributed to the fact that our BRO was made from byproducts of broccoli.

**Table 1:** Identification and Quantification of Individual Compounds Present in Broccoli Byproduct Extract (BRO)^1^

proposed compound	formula	[M]^+^ (*m/z*)	mean ± SD (mg/g)
**amino acids and derivatives**			
muramic acid/(deoxy-fructosyl)alanine	C_9_H_17_NO_7_	250	0.088 ± 0.001
fructose-aspartic acid	C_10_H_17_NO_9_	294	2.5 ± 0.2
pyroglutamic acid	C_5_H_7_NO_3_	128	3.1 ± 0.3
(deoxy-fructosyl)leucine	C_12_H_23_NO_7_	292	0.48 ± 0.02
agaridoxin	C_11_H_14_N_2_O_5_	253	1.3 ± 0.1
hydroxy-tyrosine	C_9_H_11_NO_4_	196	7.5 ± 0.2
methoxytyrosine	C_10_H_13_NO_4_	210	16 ± 1
**amino acids and derivatives/indoles and derivatives**			
benzoylaspartic acid/methyl dihydro–dihydroxy-oxo-indoleacetic acid	C_11_H_11_NO_5_	236	7.9 ± 0.4
**carbohydrates and derivatives**			
methyl-erythritol-phosphate	C_5_H_13_O_7_P	215	1.3 ± 0.2
mannonic acid/gluconic acid	C_6_H_12_O_7_	195	4.6 ± 0.3
xylonic acid/lyxonic acid/ribonic acid/arabinonic acid	C_5_H_10_O_6_	165	2.6 ± 0.3
inositol/tagatose/allofuranose/galactose	C_6_H_12_O_6_	179	0.41 ± 0.02
glucosinolate derivative	C_13_H_27_NO_9_S_2_	404	0.79 ± 0.02
galacto-heptulose/glycero-galacto-heptose/manno-heptose	C_7_H_14_O_7_	209	0.18 ± 0.02
ribosylnicotinate isomer 1	C_11_H_13_NO_6_	254	0.098 ± 0.003
dideoxy-glucopyranosyl-methyl-*ribo*-hexose	C_13_H_24_O_9_	323	0.084 ± 0.001
ribosylnicotinate isomer 2	C_11_H_13_NO_6_	254	0.55 ± 0.02
**carboxylic acids and derivatives**			
quinic acid	C_7_H_12_O_6_	191	0.02 ± 0.01
maleic acid/fumaric acid	C_4_H_4_O_4_	115	6.9 ± 0.1
malic acid/diglycolic acid	C_4_H_6_O_5_	133	6.5 ± 0.2
citric acid/isocitric acid	C_6_H_8_O_7_	191	19 ± 1
**fatty acyl glycosides/carbohydrates and conjugates**			
sarmentosin epoxide/dehydro-deoxy-acetylneuraminic acid isomer 1	C_11_H_17_NO_8_	290	2.5 ± 0.2
sarmentosin epoxide/dehydro-deoxy-acetylneuraminic acid isomer 2	C_11_ H_17_NO_8_	290	3.82 ± 0.05
sarmentosin epoxide/dehydro-deoxy-acetylneuraminic acid isomer 3	C_11_H_17_NO_8_	290	0.317 ± 0.002
**furans and derivatives**			
furoic acid	C_5_H_4_O_3_	111	1.8 ± 0.2
**hydroxybenzoic acids and derivatives**			
ethyl gallate glucuronide/syringic acid glucuronide	C_15_H_18_O_11_	373	0.23 ± 0.01
protocatechuic acid glucoside	C_13_H_16_O_9_	315	0.353 ± 0.001
hydroxycinnamic acids and derivatives			
caffeoylquinic acid/chlorogenic acid	C_16_H_18_O_9_	353	0.821 ± 0.004
caffeoylquinic acid/chlorogenic acid	C_16_H_18_O_9_	353	0.27 ± 0.02
**nucleosides**			
uridine	C_9_H_12_N_2_O_6_	243	31.7 ± 0.3
**purines**			
benzylaminopurine	C_12_H_11_N_5_	224	3.9 ± 0.1
**others**			
(dioxopyrrolidinyl)-(hydroxyethoxy)-oxopentanoate (methoxypolyethylene glycol succinimidylsuccinate)	C_11_H_15_NO_7_	272	2.2 ± 0.2
dihydroxy-dimethoxy-benzoxazinone	C_10_H_11_NO_6_	240	20 ± 1
[bis(hydroxyethyl)amino]benzeneacetic acid	C_12_H_17_NO_4_	238	6.4 ± 0.2

1 Quantification data are expressed as mg of compound/g dry extract. SD = standard deviation. Ref (11,12).

### Toxicity Evaluation *In Vivo*

**Figure 3:**
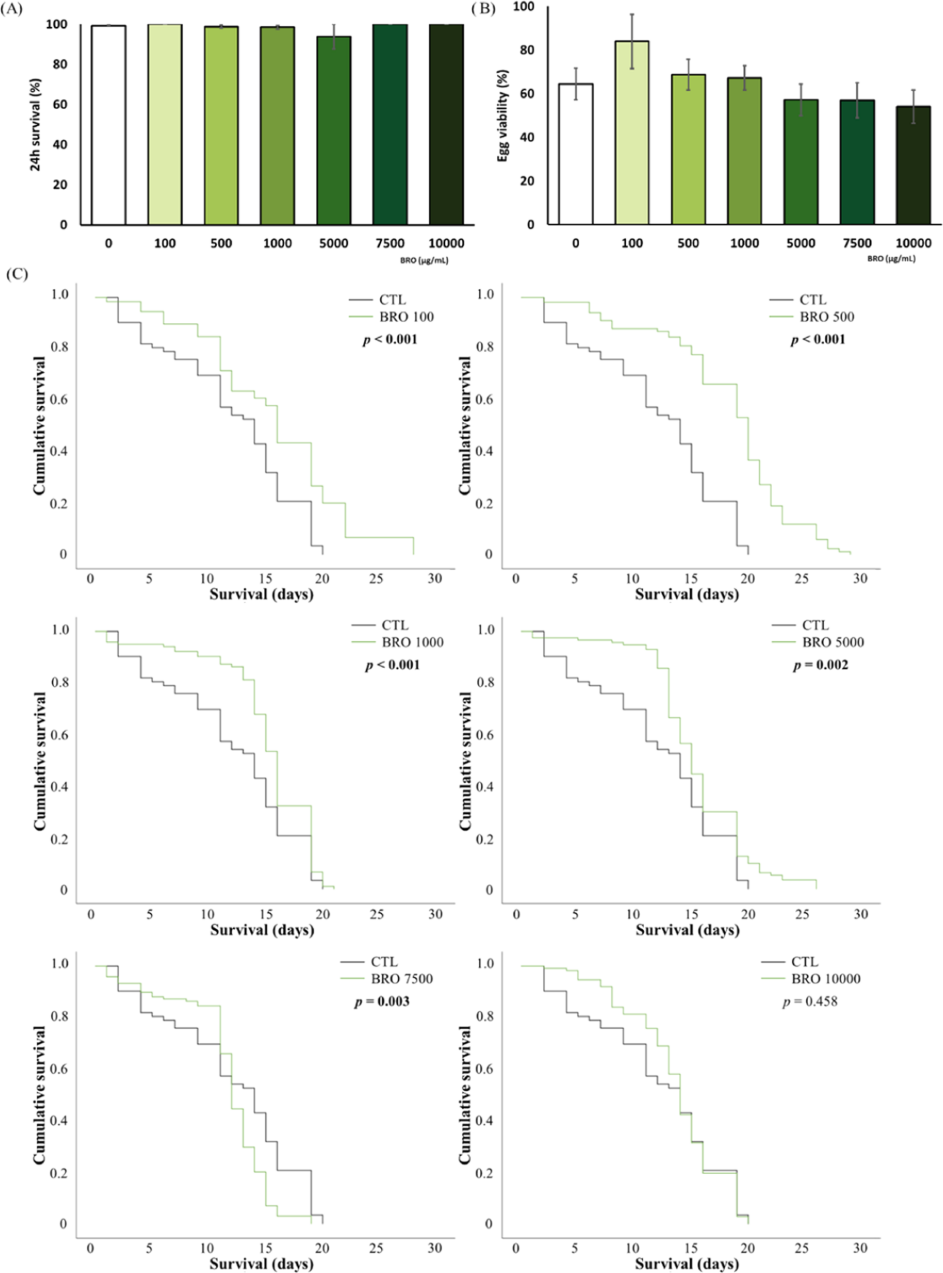
Acute- and long-term toxicity tests in *C. elegans*. (A) Lethality test in the N2 strain. (B) Egg viability test in the N2 strain. Results are expressed as mean ± SEM. (C) Long-term toxicity evaluation through the Kaplan–Meier survival curves for different concentrations of BRO (100, 500, 1000, 5000, 7500, and 10 000 μg/mL). Statistically significant differences were considered when *p* < 0.05. For survival curves, the Long-Rank test was used. CTL = control group.

Evaluation of the short-term toxic effect of the extract was assessed in the experimental model *C. elegans* by using the lethality and egg viability tests, which indicated an absence of toxicity in the concentration range 0–10 000 μg/mL (Figure 3A,B, respectively). To further explore the long-term toxicity of BRO within the same dosage range, survival curves were performed (Figure 3C). BRO did not cause a reduction in the lifespan; rather, it led to a significant increase in survival at 100, 500, 1000, and 5000 μg/mL, as supported by the Long-Rank statistical test which is particularly intriguing for the potential applications in aging. However, 7500 μg/mL reduced the worm survival although there were no significant differences between the control group and the 10 000 μg/mL group. It could be attributed to a hormetic effect of BRO treatment. The theoretical graph depicting the hormesis response is often described as a perfect “U-shape” or “inverted U-shape” in terms of dose–response effects. However, numerous biological models have shown that the hormetic response can manifest as peaks and dips before reaching the maximum hormetic effect, ultimately forming a “U-shape” or “inverted U-shape.”^28^ Notably, previous studies with sulforaphane (a molecule present in broccoli) in this model revealed similar lifespan-extending effects.^29^ No prior studies have been found evaluating the toxicity of BRO in *C. elegans* but, in agreement with our findings, Aranaz et al.^30^ reported an absence of toxicity for broccoli extract in rats. Therefore, the extract demonstrated practically no toxicity at the doses evaluated in the parameters tested, presenting an engaging prospect for potential biomedical applications. According to these results, nontoxic submaximal concentrations (100, 500, and 1000 μg/mL) were selected for further experiments.

### Effect of BRO on Redox Biology-Related Markers

**Figure 4:**
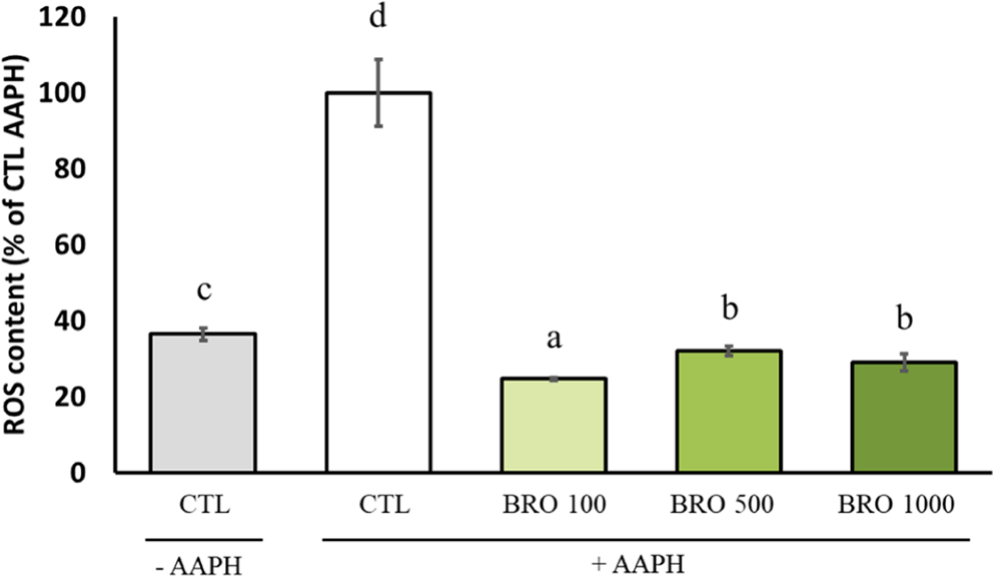
Intracellular reactive oxygen species (ROS) content after 2,2′-azobis-2-amidinopropane dihydrochloride (AAPH) damage in the N2 strain measured by the dichlorodihydrofluorescein diacetate labeling. Results are expressed as mean ± SEM. Statistically significant differences (*p* < 0.05) are represented with different lowercase letters. CTL = control group.

Experiments were conducted to investigate the influence of BRO on cellular redox status, considering its potential impact on aging and AD. The DCFDA probe was used to assess the impact of BRO on intracellular ROS content, and AAPH was employed as an oxidative stress inducer. AAPH led to higher ROS content in live worms compared with the negative control (Figure 4). The three concentrations provided protection to N2 worms against oxidation, showing less ROS content even below that of the negative control group. The most effective one was 100 μg/mL, whereas there were no differences between 500 and 1000 μg/mL. These results agree with authors who found that broccoli and its compounds exerted antioxidant effects *in vitro*(31−33) and in rodent models.^33,34^

**Figure 5:**
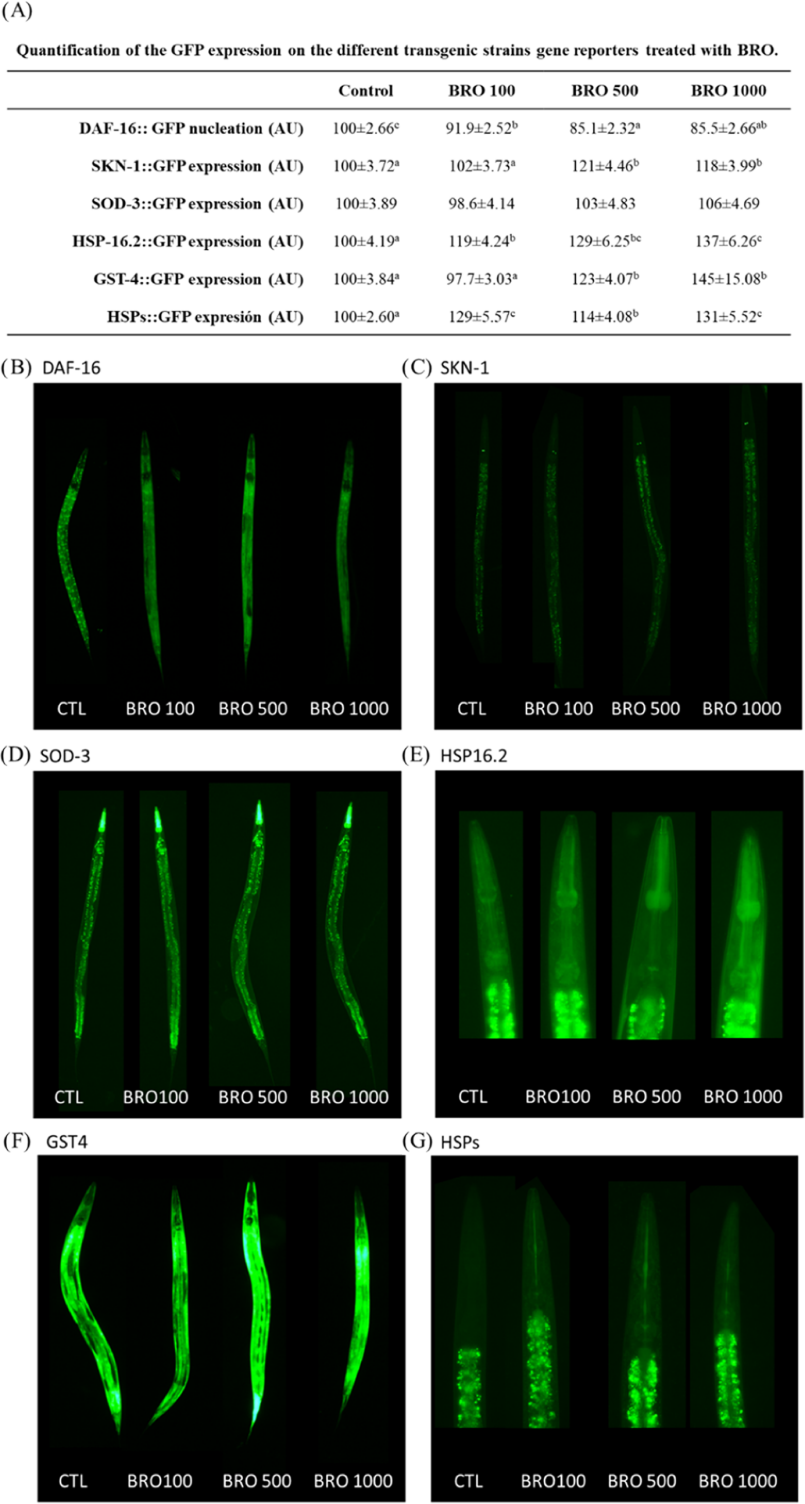
Effect of broccoli byproduct extract (BRO) at 100, 500, and 1000 μg/mL on GFP-reporter transgenic strains and illustrative images of each one. (A) Quantification of the GFP expression on the different transgenic strain gene reporters. (B) TJ356/DAF-16::GFP strain (10× magnification). (C) LD1/SKN-1::GFP strain (10× magnification). (D) CF1553/SOD-3::GFP strain (10× magnification). (E) TJ375/HSP-16.2::GFP strain (40× magnification). (F) CL2166/GST-4::GFP strain (10× magnification). (G) OS3062/HSF-1 + HSP-16.2::GFP + HSP-16.41::GFP strain (40× magnification). Results are expressed as mean ± SEM. Statistically significant differences between groups (*p* < 0.05) are represented with different lowercase letters.

The effects of BRO on the different transgenic strain GFP-reporter of genes related to redox biology at several concentrations (100, 500, and 1000 μg/mL) are collected in Figure 5A. Results indicated that the three dosages led to lower DAF-16 nucleation status, exhibiting a dose-dependent trend as shown by representative images depicted in Figure 5B. However, the expression of the SOD-3 enzyme associated with GFP, as observed in the CF1553 strain (Figure 5D), remained unaffected by any of the BRO treatments. Expression of the SKN-1 transcription factor (Figure 5C) and the GST-4 (Figure 5F) throughout the entire worm was only altered by the highest concentrations (i.e., 500 and 1000 μg/mL), resulting in higher levels. Furthermore, the three concentrations showed higher expression of several HSPs genes, as manifested by the strains TJ375 (Figure 5E) and OS3062 (Figure 5G) in the anterior pharyngeal bulb, exerting a dose-dependent effect in the case of HSP-16.2. The dose-dependent prevention in DAF-16/FOXO nucleation, a pivotal transcription factor in the insulin/insulin-like growth factor 1 signaling (IIS) pathway, is relevant. This finding could be explained by the activation of SKN-1/Nrf2 since this transcription factor can act as a negative regulator of DAF-16/FOXO.^35^ Interestingly, the expression of SOD-3, a downstream gene of DAF-16/FOXO, remained unaffected, potentially upheld by the higher expression of SKN-1/Nrf2 mediated by BRO treatment. Consistent with this observation, previous studies have shown an upregulation of Nrf2 expression in cells treated with sulforaphane^31^ or broccoli juice^32^ as well as in rats treated with sulforaphane-enriched broccoli sprouts.^36^ Furthermore, SKN-1/Nrf2 could be mediating the higher expression of GST-4 and HSPs, which are also its downstream genes. In line with our results, some researchers have proved that overexpression of HSPs is involved in the mild-stress response in *C. elegans.*(37,38) HSPs are generally recognized as important pieces of adaptive responses and their expression is often correlated with the presence of several stressors. However, it is common for some bioactive compounds to present hormetic effects. In our case, BRO treatment may be triggering low/mild stress that results in the activation of multiple stress-responsive genes for the organism’s benefit. The low/mild stress induced by BRO may be initiating an adaptive cellular response that prepares worms for subsequent severe stress, conferring thus stress resistance and providing beneficial effects.

In agreement, sulforaphane treatment increased the levels of HSP70 in a transgenic mouse model of AD, which was observed to be related to clear the accumulation of Aβ and tau using primary cell culture.^39^ Regarding the enzyme GST-4, experimental evidence has shown that GSTs also participate in glucosinolate catabolism,^40^ so that may be another reason to explain the observed higher expression level. Therefore, BRO appears to act as an antioxidant agent and a modulator of the redox biology in worms mainly through the activation of the SKN-1/Nrf2 transcription factor. These findings hold significant promise for potential applications of BRO treatments in physiological conditions involving alterations in redox biology, such as aging, or in diseases like AD.

### Effect of BRO on Alzheimer’s Disease Markers

*In vitro* AChE inhibitory capacity as well as *in vivo* effects on Aβ-related toxicity and influence on Tau-associated proteotoxicity were evaluated as AD markers. Concerning AChE, IC_50_ for BRO was 2869 μg/mL, calculated from a dose–response curve and using the equation “*y* = −2 × 10^–06^*x*^2^ + 0.026*x* – 8.1345.” IC_50_ for PHY was 0.009 μg/mL, calculated from the equation “*y* = −223565 *x*^2^ + 8817.3*x* – 10.817”. Alterations in the cholinergic system are associated with AD pathology. AChE and BChE play a role in degrading the neurotransmitter Ach, and their increased activity appears to promote Aβ fibril formation. The levels of cholinesterases are increased in the brain of AD patients, resulting in a deficit in cerebral cholinergic neurotransmitters ultimately leading to memory loss and other cognitive symptoms.^41^ Thus, compounds able to inhibit them could be an interesting approach for the treatment of the symptomatology of this disease. Scientific literature on the effects of florets or stems of broccoli in this context is limited, but other parts of the vegetable such as the leaves have been evaluated in this regard. Data from *in vitro* experiments revealed the AChE inhibitory activity of the chloroform fraction of broccoli leaves extract^33^ and of different isothiocyanate compounds.^42^*In vivo* studies have also confirmed this effect.^33,34,36^ Nevertheless, the IC_50_ of byproduct was much higher than that of the positive control PHY. PHY is a potent parasympathomimetic alkaloid known to inactivate ChE and increase Ach levels at cholinergic synapses in the nervous systems.^43^ Therefore, the AChE inhibitory activity exhibited by BRO treatment should be considered relatively modest when compared with drugs specifically designed for that purpose.

**Figure 6:**
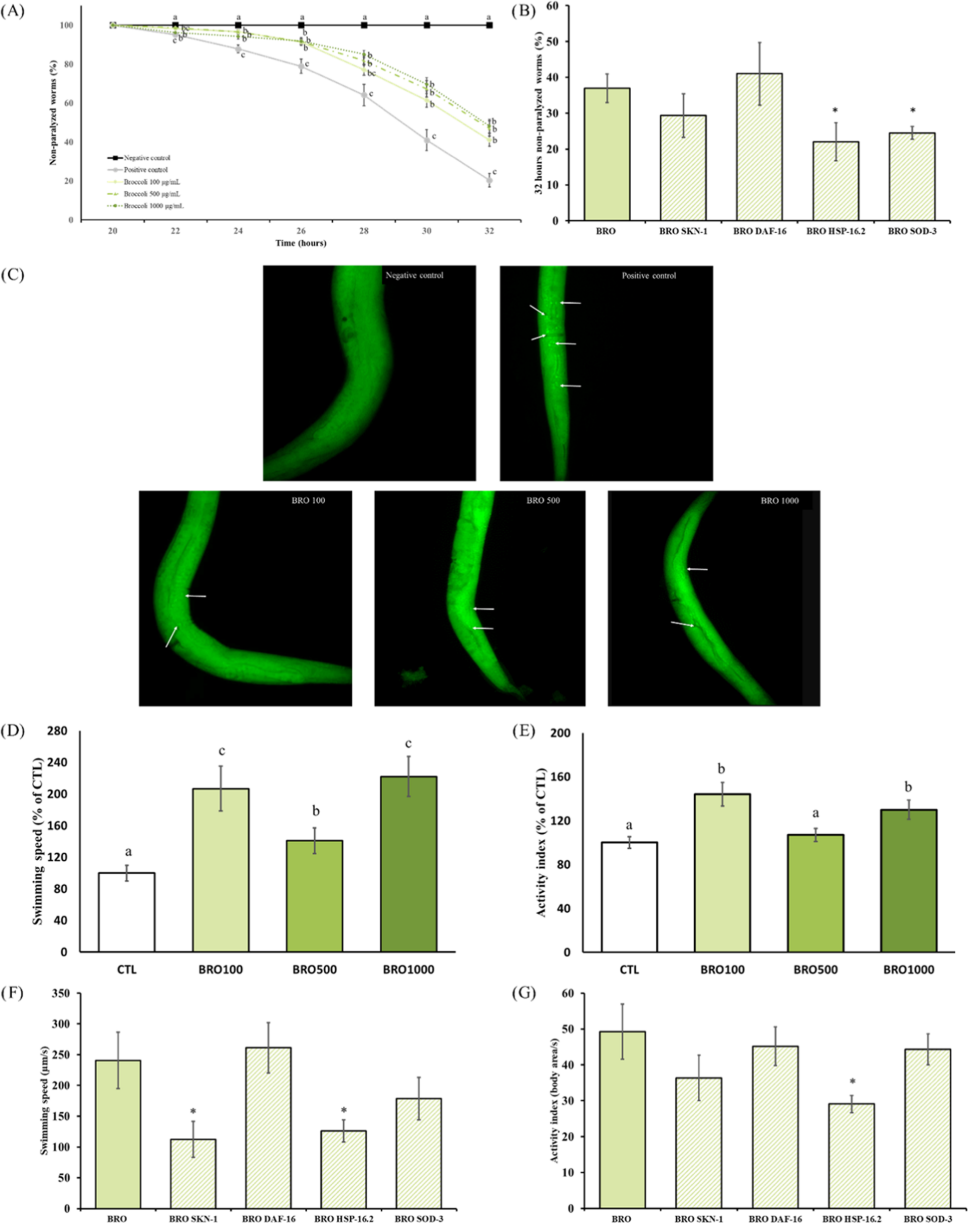
Effect of the broccoli byproduct extract (BRO) on the amyloid-β -induced paralysis phenotype, tau-related proteotoxicity, and visualization of amyloid β aggregation, and the effect of the different RNAi (SKN-1/NRF2, DAF-16/FOXO, HSP-16.2, and SOD-3) in the transgenic strains CL4176 and BR5706. (A) Paralysis curve represented as nonparalyzed CL4176 worms (%) from 20 h until 32 h after the temperature upshift for 100, 500, and 1000 μg/mL. (B) Nonparalyzed CL4176 worms (%) at 32 h after the temperature upshift treated with BRO 100 μg/mL and with or without RNAi. (C) Representative images of the thioflavin T staining in CL4176 worms collected 26 h after the temperature upshift (40× magnification) for negative and positive controls, and treated worms with BRO 100, 500, and 1000 μg/mL. White arrow shows the Aβ aggregated. (D, E) Effects of BRO 100, 500, and 1000 μg/mL on locomotive parameters in the transgenic strain BR5706: Swimming speed (D) and activity index (E). (F, G) Influence of the different RNAi on swimming speed (F) and activity index (G) for BRO 100 μg/mL. Results are expressed as mean ± SEM. Statistically significant differences between groups (*p* < 0.05) are represented with different lowercase letters. Significant differences (*p* < 0.05) between the treatment with and without the RNAi are represented by *.

The amyloid cascade hypothesis, a widely accepted theory in AD, posits that Aβ deposition initiates a sequence of events, including microglial activation, inflammatory response, and reactive astrocytosis, ultimately leading to neuronal dysfunction.^44^ Thus, the influence of BRO on the Aβ toxicity was elucidated by the paralysis test, which was carried out using the transgenic strain CL4176. These worms express the human Aβ_1–42_ peptide in muscle cells, leading to a paralysis phenotype when the incubation temperature is raised from 16 to 25 °C. The assessed concentrations of BRO (100, 500, and 1000 μg/mL) demonstrated a clear delay in paralysis in treated worms (Figure 6A). Significant differences between the control and treated groups were observed from 22 h after the temperature increase until the last reading, with no differences between dosages. At the end reading point, over 40% of the treated worms remained unparalyzed, compared with 20% in the control group. These results were corroborated by thioflavin T staining, which specifically binds to Aβ aggregates. The positive control displayed a large amount of Aβ deposits as evidenced by the presence of numerous green shiny dots (Figure 6C). In contrast, the negative control showed a total absence of such aggregates, and the BRO treatments manifested a significantly lower amount compared with the positive control. There are no data on the effect of broccoli or its byproducts on Aβ aggregation, although positive results have been demonstrated for sulforaphane in transgenic mice.^45^

Accumulation of hyperphosphorylated tau protein is another hallmark of AD, and studies have indicated that Aβ and tau act synergistically to promote the pathological progression of the disease and neuronal loss.^44^ Therefore, reducing the accumulation of both proteins has been considered one of the most promising therapeutic approaches for AD. *C. elegans* transgenic strain BR5706 exhibits accelerated aggregation of insoluble Tau in neurons, leading to defects in the nervous system and altered movements. WormLab station and associated software were used to investigate swimming locomotive behavior. Swimming speed measured the velocity of the animals during a two-stroke interval, while activity quantified the brush stroke normalized by the time taken to perform the two strokes. Treatment for 72 h with 100 and 1000 μg/mL BRO led to higher swimming speed and activity than control (Figure 6D,E). The concentration of 500 μg/mL was less effective than the other two but still demonstrated a significant difference compared with the control group, particularly for the swimming speed parameter. These findings indicate the potential of BRO to ameliorate the effects of tau protein aggregation in this *C. elegans* model. Scientific literature about the effect of broccoli or its compounds on tau protein is quite scarce, but again, sulforaphane cleared the accumulation of tau in a primary cell culture^39^ and the hyperphosphorylation in a transgenic mice model.^45^ Other natural products have also demonstrated a positive effect on Aβ and Tau toxicity in this experimental model, such as strawberry,^14,22^ avocado honey,^23^ and oleuropein-^16^ and hydroxytyrosol-^17^ rich olive leaves extract.

Most of the studies investigating mechanisms underlying the effect of natural compounds against Aβ-induced paralysis or tau-induced movement impairment in *C. elegans* focus on the pathways addressed in previous sections: those related to oxidative stress, heat shock response, insulin signaling cascade, and redox homeostasis.^46^ According to that, RNAi for those pathways or proteins was applied to the paralysis and movement analysis, with the lowest effective concentration of BRO (100 μg/mL) selected for the tests. RNAi was administered to the worms by feeding through the *E. coli* HT115 that contained dsRNA for *skn-1*, *daf-16*, *hsp-16.2*, and *sod-3* genes. Figure 6B shows RNAi paralysis assay of CL4176 worms for BRO 32 h after incubation temperature rising. The inhibition of *sod-3* and *hsp-16.2* genes led to a significantly lower percentage of nonparalyzed worms. However, the paralysis was not modified by the administration of RNAi for *skn-1* or *daf-16*, indicating that these genes were not directly associated to the observed benefits. On the other hand, the inhibition of *skn-1* and *hsp-16.2* led to a lower swimming speed in BR5706 worms leading to nonsignificant differences when compared with the nontreated group. However, only *hsp-16.2* was involved in the BRO-induced higher activity index (Figure 6F,G). These findings provide valuable insights into the specific genes that might mediate the beneficial effects of BRO in attenuating Aβ toxicity and Tau proteotoxicity in *C. elegans*. The key protein common to the positive effects of BRO on Aβ and Tau toxicity was HSP-16.2. HSP-16.2 plays a crucial role in maintaining protein homeostasis involved in highly conserved stress responses that prevent protein mismanagement. In *C. elegans*, HSP-16.2 directly interacts with Aβ peptide, disrupting its oligomerization and reducing the formation of toxic species.^47,48^ Furthermore, SOD-3 was related to the protective effect against Aβ toxicity, while the SKN-1 factor was associated with the effect on tau toxicity. RNAi results agree in part with experiments on redox biology-related transgenic strains such as the participation of HSP-16.2. However, the SKN-1/Nrf2 was not modified by 100 μg/mL in baseline situations (GFP-reporter strains) but it was involved in the AD benefits, same as observed for SOD-3, which might indicate that those proteins are activated under AD. It is conceivable that, in strains under normal conditions, certain proteins or pathways remain deactivated because they are unnecessary in that context, hence BRO does not exert any effect. However, the presence of Aβ or tau proteins may induce stress, demanding the activation of those specific pathways, creating an environment where BRO can effectively modulate the stress with increased activity compared with normal conditions. This scenario aligns with other findings reported in another study in *C. elegans* using oleuropein-rich olive leaves extract.^16^

### Effect of BRO on Oxidative Stress Markers in Aging

**Figure 7:**
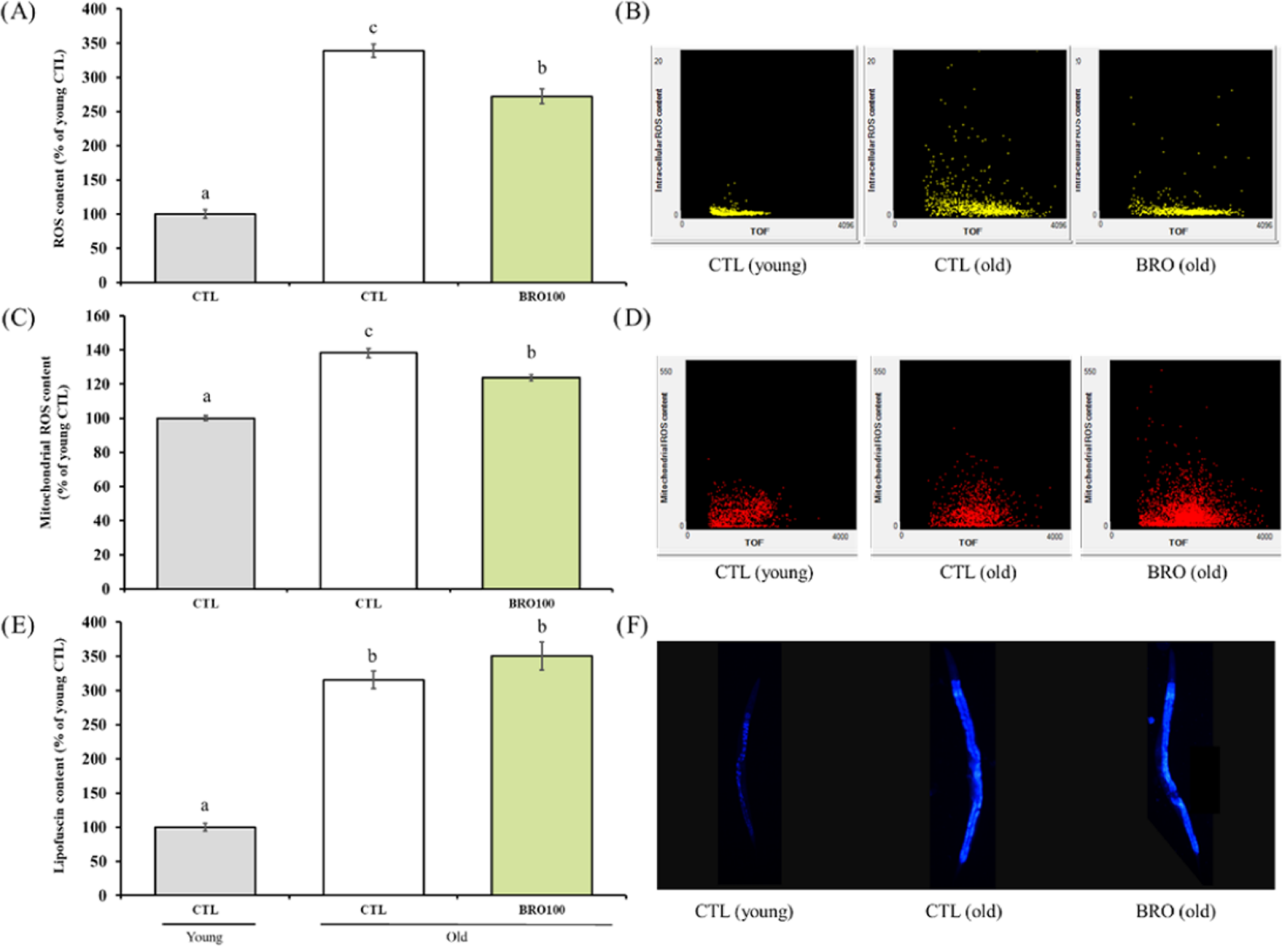
Effect of broccoli byproduct extract (BRO) 100 μg/mL on oxidative stress markers on aging (young worms = 5 days old; old worms = 12 days old). (A) Intracellular reactive oxygen species (ROS) content measured by the DCFDA technique. (B) Representative dot plots panels of intracellular ROS content vs TOF (time-of-flight; worm size) of yellow fluorescence intensity extracted from flow cytometer software. (C) Mitochondrial ROS content measured by Mitotracker staining. (D) Representative dot plots panels of mitochondrial ROS content vs TOF of red fluorescence intensity extracted from flow cytometer software. (E) Lipofuscin content. (F) Representative images of lipofuscin for each group (10× magnification) obtained by using an epi-fluorescence microscope with a DAPI (blue) filter. Lowercase letters, when different, represent statistically significant differences (*p* < 0.05). Results are expressed as mean ± SEM. CTL = control group.

Considering the social and health importance of aging and since AD is an age-related disease, in the present study, the effect of BRO was preliminary assessed concerning this physiological situation. One of the features of aging is the increase in ROS production with subsequent oxidative damage.^49^ To test this hypothesis, two different populations of N2 worms were used: 5-day-old animals as the young control group, and 12-day-old animals as both, control and treated old nematodes. The age and synchronization of the worms were adjusted to ensure the same reading point for both populations. DCFDA and Mitotracker probes were used to test the effect of BRO on intracellular and mitochondrial ROS content, respectively. Aging led to significantly higher values for intracellular (Figure 7A,7B) and mitochondrial (Figure 7C,7D) ROS, standing out the total content since it was three times higher in the old control group than in the young counterparts. BRO at 100 μg/mL exhibited substantial protection against the age-related rise in ROS levels in both probes. Specifying, BRO treatment led to a less total and mitochondrial content by approximately 20 and 11%, respectively, when compared with the aged control group. These results demonstrated the ROS theory of aging under the present model showing the antiaging potential of our broccoli byproduct. Lipofuscin, an indicator of both oxidative stress and aging in *C. elegans*, is a native autofluorescent pigment formed by cross-linked polymeric substances due to oxidative processes that accumulate progressively over time, particularly in lysosomes and gut granules within the intestine. Its measurement is used as a marker for evaluating healthspan in aging, being one of the widely studied age-dependent biomarkers in nematodes.^50^ Results on lipofuscin are presented in Figure 7E,F. Aging led to a higher lipofuscin content, with values of control aged worms being more than three times higher than their young counterparts. In this parameter, BRO did not exhibit any significant effect. That observation could be attributed to the inherent nature and implications of the lipofuscin pigment. The formation and accumulation of lipofuscin lead to a variety of defects in cellular function and homeostasis. It is associated with progressive decline in lysosomal function and disruptions in both phagocytosis and autophagy processes, which subsequently affect numerous cellular activities. Lipofuscin acts as proteasome inhibitor by directly binding on proteasome complexes. Consequently, processes unrelated to ROS are also linked to lipofuscin accumulation, including autophagy.^51,52^ Therefore, that kind of process may be altered in aged worms, which may not be modulated by BRO, explaining the noneffect observed in lipofuscin content. No data on broccoli or its byproducts have been found; however, other treatments, such as strawberry extract, partially prevented the accumulation of this pigment at the same concentration as BRO, probably mediated by anthocyanins.^22^

While this research provides valuable insights, the specific molecules responsible for the observed effects remain unclear. However, drawing from literature, some of the primary compounds found in the extract, such as uridine, hydroxycinnamic acids, or the characteristic glucosinolate, could account for these positive effects. For instance, studies have demonstrated that glucosinolate-rich broccoli sprouts protected against oxidative stress in humans,^53^ and its derivate isothiocyanates interfered with the molecular cascades of AD pathogenesis, preventing functional loss in neurons.^54^ Furthermore, chlorogenic acid reduced the aging-induced ROS production in *C. elegans* and increased worms’ lifespan and healthspan.^55^ Caffeoylquinic acid reversed cognitive deficits in AD model mice,^56^ and both phenolic acids exhibited neuroprotective properties by inhibiting AChE and BChE activities as well as preventing oxidative stress-induced neurodegeneration in rats.^57^ Additionally, lower uridine levels have been associated with AD progression,^58^ and the administration of a uridine prodrug reduced cognition impairments, tau phosphorylation, lipid peroxidation, ROS levels, and mitochondrial DNA damage.^59^

In conclusion, the extract assayed in the present study, derived from broccoli byproducts, exhibited significant antioxidant activity attributed to its profile and content on bioactive compounds, demonstrated both *in vitro* and in the *C. elegans* model. *In vivo*, this effect could be mediated by the modulation of several markers associated with redox biology, highlighting the activation of the transcription factor *skn-1*/*Nrf2* and its downstream genes *gst-4* and *hsps.* Regarding AD features, the extract demonstrated to possess a moderated AChE inhibitory capacity *in vitro* and the ability to prevent *in vivo* the Aβ- and tau-induced proteotoxicity in transgenic strains via SOD-3 and SKN-1, respectively, and HSP-16.2 for both parameters. Results from the RNAi tests mostly agreed with those observed in GFP-reporter transgenic strains, with all of the benefits being independent of DAF-16/FOXO pathway. Furthermore, a preliminary study on aging indicated that the extract effectively prevented intracellular and mitochondrial ROS accumulation in aged worms and extending at the same time their lifespan. These findings pave the way for further exploration of underlying mechanisms in the aging context. Therefore, this broccoli byproduct extract displayed the ability to modulate redox biology, influencing positively then on aging and AD. Altogether, results hold significant importance as they demonstrate the remarkable efficacy of the broccoli byproduct extract. It is noteworthy that the extract is derived from a broccoli byproduct rather than directly from the broccoli itself and it supports the use of broccoli byproducts for nutraceutical or functional food development. Doing this would not only manage vegetable processing waste but also enhance the productivity and sustainability of the broccoli crop while providing significant health benefits.

## Abbreviations

Aβ: amyloid β peptide
ABTS: 2,2′-azino-bis(3-ethylbenzothiazoline-6-sulfonic acid)
AChE: acetylcholinesterase
AD: Alzheimer’s disease
AAPH: 2,2′-azobis-2-amidinopropane dihydrochloride
BRO: broccoli byproduct extract
C. elegans: Caenorhabditis elegans
DAF: dauer formation
DCFDA: dichlorodihydrofluorescein diacetate
DE: dry extract
DPPH: 2,2-diphenyl-1-picryl-hydrazyl-hydrate
DTNB: 5,5′-dithiobis(2-nitrobenzoic acid)
E. coli: Escherichia coli
FRAP: ferric reducing antioxidant power
FOXO: Forkhead box transcription factors class O
FUDR: 5-fluoro-2′deoxyuridine
GFP: green fluorescent protein
GST-4: glutathione S-transferase 4
HSP: heat shock protein
IC_50_: half-maximal inhibitory concentration
IPTG: isopropyl β-d-1-thiogalactopyranoside
IIS: insulin/insulin-like growth factor 1 signaling
NGM: nematode growth media
Nrf2: nuclear factor erythroid 2-related factor 2
PHY: physostigmine
RNAi: RNA interference
ROS: reactive oxygen species
SKN-1: transcription factor skinhead-1
SOD: superoxide dismutase
TAC: total antioxidant capacity
TE: trolox equivalent
TFC: total flavonoid content
TPC: total phenolic content
